# Sella Turcica Abnormalities, Dental Age and Dental Abnormalities in Polish Children

**DOI:** 10.3390/ijerph181910101

**Published:** 2021-09-26

**Authors:** Tomasz Jankowski, Maciej Jedliński, Krzysztof Schmeidl, Katarzyna Grocholewicz, Joanna Janiszewska-Olszowska

**Affiliations:** 1 Private Practice Dental Clinic Jankowscy, 68-200 Żary, Poland; tomaszjankowski1905@gmail.com; 2 Department of Interdisciplinary Dentistry, Pomeranian Medical University in Szczecin, 70-204 Szczecin, Poland; krzysztof.schmeidl@gmail.com (K.S.); katgro@pum.edu.pl (K.G.); jjo@pum.edu.pl (J.J.-O.)

**Keywords:** sella, cephalometry, radiography, panoramic, hypodontia, cuspid

## Abstract

The frequency of some sella turcica abnormalities on cephalometric radiographs is age related. Chronological age might not overlap with growth; however, no studies could be found on the association between sellar morphology and dental age. Although an association exists between sella turcica bridging and dental abnormalities, no studies have been found correlating sellar abnormalities other than bridging with dental abnormalities. The aim of this study was to find any correlations between sella turcica abnormalities and dental age or dental abnormalities. Methods: Lateral cephalograms and panoramic radiographs of 206 children aged 6–15 years were analyzed for sela turcica abnormalities, Demirijan dental age, and dental abnormalities. Results: The prevalence of dental abnormalities in patients with sela turcica abnormalities was 16.98%, while in those with normal sella, it was 3%. The differences between dental and chronological age were higher in patients with sella turcica abnormalities (*p* = 0.002). Dental abnormalities were more prevalent (*p* = 0.001) in patients with sellar abnormalities other than sellar bridging than in those with sellar bridging or normal sella. Conclusions: Sella turcica abnormalities are correlated with delayed dental age. Dental abnormalities are more frequent in patients with sellar abnormalities. Dental abnormalities are less frequent in subjects with sellar bridges compared to those with other sellar abnormalities.

## 1. Introduction

The sella turcica is a superior saddle-shaped depression located on the intracranial surface of the sphenoid bone [1]. This structure contains an important anatomical reference in orthodontics: the S-point, located centrally in the sella region, partly because the contour of the anterior wall is used in the assessment of craniofacial growth [2]. The sella turcica contains the pituitary fossa, which hosts the pituitary gland and consists of three lobes: the anterior lobe (adenohypophysis), intermediate lobe, and posterior lobe (neurohypophysis) [3]. Pituitary gland pathology may result in sella turcica shape malformation, or in the altered regulation of glandular hormones (prolactin, growth hormones, thyroid-stimulating hormones, or follicular stimulating hormones) [3].

Axelsson et al. [4] described a normal sella turcica anatomy and defined five types of sellar abnormalities: oblique anterior wall, sella turcica bridging, double contour of the floor, irregularity (notching) in the posterior part of the dorsum sellae, and pyramidal shape of the dorsum sellae. Kucia et al. [5] added three other variants of sellar dysmorphology to the classification: hypertrophic posterior clinoid process, hypotrophic posterior clinoid process, and oblique contour of the floor. The most commonly investigated anomaly of sella turcica is sella turcica bridging [4,5,6,7,8,9,10,11,12,13,14,15,16,17,18,19,20,21,22,23,24,25,26,27]. Bridging is described as a fusion of anterior and posterior clinoid processes [6]. Becktor et al. [6] described two types of sella turcica briding: A—manifest, ribbon-like fusion; B—extension of the anterior and/or the posterior clinoid process (thin fusion anteriorly, posteriorly or in the middle).

In cephalometric studies including healthy individuals, complete sella turcica bridging appears from 1.10% to 11.67% [4,5,7,8,9,10,11,12,13,14,15,16,17,18,19,20,21]. Kucia et al. [5] proved that compared to those with normal sella, craniofacial morphology in patients with sella turcica abnormalities was characterized by significantly higher angles of incisor inclination and a more retruded mandibular alveolus. Similarly, Motwani et al. [18] stated that sella turcica abnormalities are related to malocclusion.

A higher prevalence of sella turcica bridging has been reported in subjects with dental abnormalities (6.45–33.30%) [13,16,22,23,24,25,26,27]. A recent meta-analysis [28] confirmed a clear association between dental abnormalities and sella turcica bridging on cephalometric radiographs. However, no studies have been found that refer to any possible correlation between sella turcica abnormalities other than sellar bridging and dental abnormalities.

There are reports in the literature that the frequency of some sellar abnormalities is correlated with age, such as Caderberg et al. [11] or Arcos-Palomino and Ustrell-Torent [29]. It is known that chronological age does not always overlap with growth rates, including bone age, dental age, assessment of secondary sex characteristics, morphological age, and mental age [30].

Verma et al. [31] classified dental age estimation into three categories: morphohistological methods, radiological methods, and biochemical methods. In orthodontics, radiological methods including that of Demirjian, which is based on eight stages of tooth development of the seven left mandibular teeth (Demirijan and Goldstein) are very popular. Dental age describes the development of permanent dentition, and is less influenced by external factors, such as nutrition and hormone metabolism. Therefore, the assessment of dental age is used in orthodontics when planning the treatment of young patients [29]. It has been found that dental age is delayed in subjects with ectopic maxillary canines [32,33].

However, no studies could be found investigating associations between the morphology of sella turcica on cephalometric radiographs and dental age.

Therefore, the aim of this study was to find out whether sella turcica types are associated with dental age or the presence of dental abnormalities.

## 2. Materials and Methods

### 2.1. Study Material

After obtaining permission, 628 lateral cephalograms were analyzed for the following inclusion criteria:

- Good visibility of sella turcica;
- Recorded date of birth and image obtaining date;
- Available panoramic radiograph performed on the same day, with good visibility of the dentition.

The exclusion criteria were as follows:

- Craniofacial deformities;
- Presence of severe systemic diseases.

Finally, lateral and panoramic radiographs of 206 patients meeting the criteria at ages ranging from 6 to 15 years were selected. One hundred and six lateral cephalograms with sella turcica anomalies were defined as the study group. The other 100 cephalograms with normal anatomy of sella turcica served as the control group. The study was conducted in the years 2019–2021 at the Department of Interdisciplinary Dentistry of Pomeranian Medical University in Szczecin. There were no participants in the study. The study was based on anonymized records from the radiological department. Panoramic radiographs and cephalograms were taken using the same equipment: Cranex 3DX (Soredex, Kavo Imagining GmbH, Berlin, Germany). The study was exempted from ethical approval by the ethical committee of Pomeranian Medical University in Szczecin (declaration reference no. = KB-012/104/09/2021/Z)

**Figure 1:**
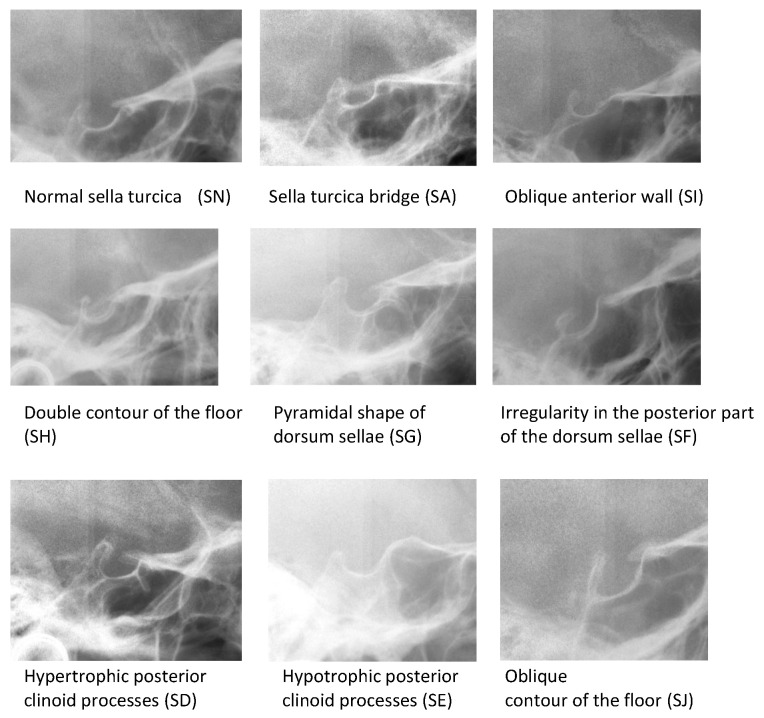
Types of sella turcica.

Sella turcica shapes were classified according to previous studies [4,5], as presented in Figure 1, into 9 groups, including normal sella turcica (SN) and sella turcica anomalies:

- Sella turcica bridging (SA, SB, SC);
- Hypertrophic posterior clinoid process (SD);
- Hypotrophic posterior clinoid process (SE);
- Irregularity (notching) in the posterior part of the dorsum sellae (SF);
- Pyramidal shape of the dorsum sellae (SG);
- Double contour of the floor (SH);
- Oblique anterior wall (SI);
- blique contour of the floor (SJ).

Subsequently, sella turcica bridging was classified into three subgroups [7,13]:

- Type A: ribbon-like fusion (SA);
- Type B: extension of the anterior and/or posterior clinoid process (SB);
- Incomplete bridge defined as partial calcification of interclinoid ligament (SC).

Dental age estimation was performed on panoramic radiographs according to Demirjian’s method [34]. Maturity scores, given according to the developmental criteria of each of the seven left permanent teeth of the mandible, were summed to obtain an overall maturity score, which was subsequently converted into a dental age using published conversion tables [34]. Six subjects in the study group had agenesis of one or more teeth, which is required in dental age determination. Therefore, the dental ages of 100 patients in the study group and 100 patients in the control group were estimated. Dental age was compared with chronological age. A Wilcoxon signed-rank test was used to scan the accuracy of Demirjian’s technique. All the radiographs were examined by the first author.

The following dental anomalies were searched for on panoramic radiographs: palatally displaced canine (PDC), hypodontia, hyperdontia, transposition, and impacted teeth.

Data were examined according to gender and three age groups (up to 9 years, 10–11 years, and over 11 years). The division into age groups was conducted so that each age group had a similar number of patients.

### 2.2. Error Study

Twenty randomly selected subjects were re-examined by the same author 3 months after initial tracings. The intraclass correlation coefficient (ICC) and Dahlberg’s coefficient regarding maturity scores in Demirjian’s method were calculated to assess the agreement between examinations. The accordance between two analyses of sella turcica type was verified using Cohen’s kappa.

### 2.3. Statistical Analysis

Associations between sella turcica type and dental age were analyzed using the Mann–Whitney and Kruskal–Wallis tests. To assess the correlation between sella turcia type and dental anomalies, a Chi-square test with Yate’s correction and Fisher’s exact test were used.

The level of the significance was considered as *p* < 0.05. Analysis was made using program R, version 4.0.3. [35]. R is a language and environment for statistical computing (R Foundation for Statistical Computing, Vienna, Austria. URL https://www.R-project.org/, accessed on 8 June 2021).

## 3. Results

The intraclass correlation coefficient (ICC) and Dahlberg’s coefficient regarding maturity scores in Demirjian’s method showed a very high level of agreement between repeated examinations. Moreover, a very high accordance was found between two analyses of sella turcica type (Cohen’s kappa).

The distribution of the study group according to chronological and dental age is presented in Table 1. The mean difference between chronologic age and dental age was –18 months.

The distribution of all types of sella turcica in the present study is presented in Table 2. Normal morphology of the sella turcica was found in more than 50% of patients.

The distribution of dental abnormalities in the study and control groups is presented in Table 3. In the study group of patients with sella turcica abnormalities, there were 20 dental abnormalities diagnosed. Two patients from the study group presented with two types of dental abnormalities. In the control group, dental abnormalities were found in three patients. Thus, the prevalence of dental abnormalities in the study group was 16.98%, while in the control group with normal sella turcica anatomy, it was 3%. The most prevalent dental abnormality in the study group was hypodontia (9.43%). Nine out of ten cases of hypodontia were found in sella turcica abnormalities, excluding bridges (SA, SB, and SC). Hypodontia affected upper lateral incisors and upper or lower premolars.

No correlation was found between sella turcica type on cephalometric radiographs and dental age or Demirjian’s maturity scores in the study group of patients aged 6 to 15 years. On the other hand, the difference between dental and chronological age was significantly higher in patients with sella turcica abnormalities (*p* = 0.002) compared to those with a normal sella. Thus, the dental age of subjects with sellar abnormalities was delayed according to chronological age compared to the group with normal sella. When comparing sella turcica bridge to other sellar abnormalities, dental abnormalities were more prevalent (*p* = 0.001) in patients with sellar types SD, SF, SG, SH, and SJ than in those with SN, SA, SB, and SC. Additionally, they were least prevalent in those with normal sella (SN).

## 4. Discussion

Numerous studies have been published referring to abnormalities of sella turcica on cephalometric radiographs [4,5,6,7,8,9,10,11,12,13,14,15,16,17,18,19,20,21,22,23,24,25,26,27,29]. Differences were found in the prevalence of sella turcica bridging. The normal sella turcica morphology reported in more than 50% of the patients in the present study was a similar percentage to that reported by Kucia et al. [5], but lower than the 66% reported by Alkofide [7] and the 68% reported by Axelsson et al. [4]. The higher prevalence of sella turcica abnormalities in the present study (compared to Alkofide and Axelsson et al.) resulted from expanded diagnostic criteria used according to a previous study by Kucia et al. [5].

Concerning the appearance of sellar bridging on cephalometric radiographs, a bridge of the sella turcica on a panoramic radiograph may represent a union or overlapping. Moreover, Carstens [9] found differences related to horizontal head position. The morphology can be verified on 3D radiographs; however, these cannot be used for routine orthodontic diagnostic measures. Evidence on sella turcica abnormalities on CBCT is scarse [26,36]. The main indication for CBCT is imaging impacted teeth [31], including third molars [26] as well as odontogenic bony lesions [37]. The scans are usually restricted to the area of dental pathology and do not comprise sella turcica. No data on sella turcica morphology in a control group without any dental pathology can be found in any study.

Demirjian’s method of dental age estimation was developed in 1973 [38] and further improved in 1976 [34]. It was based on the assessment of the crown and root stage development of the lower mandibular teeth on panoramic radiographs of 1446 boys and 1428 girls (increased to 2407 boys and 2349 girls in the newer study) from the French-Canadian population, aged 2–20. Age estimation is used for the forensics identification of unknown bodies [30]. In recent years, there has been an increase in global migration. Therefore, age estimation is becoming relevant for individuals without valid documents to ascribe their authentic age in the course of civil, criminal, asylum, or old-age pension proceedings [30]. There are reports in the literature indicating that chronological age calculated with Demirjian’s method is overestimated, due to the acceleration of tooth development or ethnic differences. According to a meta-analysis by Jayaraman et al. [39], the Demirjian dataset overestimated the age of females by 0.65 years (−0.10 to +2.82 years) and males by 0.60 years (−0.23 to +3.04 years). A recent meta-analysis of studies performed on the Indian population revealed that compared to the chronological age, Demirjian’s method overestimated the dental age by nearly 5.5 months [40]. These results are inconsistent with the present study, where dental age was overestimated by a mean of18 months. The high diversity may be caused by the different age ranges and structures of the patients examined. In the present study, the age range was from 6 to 15 years. A high percentage of patients in the pubertal stage of development might be associated with high individual differences in the onset of maturation stages.

In the present study, 16.98% of patients with sella turcica abnormalities had dental abnormalities. Scarce data that could be used for the discussion have been found in the available literature that refers to the prevalence of dental abnormalities and compares patients with normal sella to those with different sella turcica abnormalities. Dixit et al. [17] compared a study group with sella turcica bridging to a control group with normal sellar morphology, and showed significant differences relating to the occurrence of dilaceration, microdontia, persistent deciduous teeth, supernumerary teeth, hypodontia, impacted teeth, buccally or palatally erupted teeth, rotated teeth, and spacing. This is in accordance with the present study, where subjects with sella turcica bridging had higher frequencies of dental abnormalities than subjects in the control group. Moreover, the present study showed a higher prevalence of dental anomalies in other sella turcica types (SD–SJ).

In previous studies, the prevalence of sella turcica abnormalities was analyzed in groups with dental abnormalities and compared with healthy subjects [13,16,22,23,24,25,26,27,29]. Leonardi et al. showed that patients with a palatally displaced canine (PDC) or missing mandibular second premolar had a higher frequency of sella turcica bridging [13]. Scribante et al. widened the spectrum of dental abnormalities (palatally and buccally impacted canines, upper lateral incisors, lower second premolar agenesis, and hyperdontia conditions) and drew similar conclusions [22]. Furthermore, subjects with calcifications in the region of sella are at potential risk of developing dental transposition [16]. Similar results were reported by other authors [23,24,25,27], who investigated correlations between sella turcica bridging and dental abnormalities on lateral and panoramic radiographs. Thus, most authors agree that abnormal sella turcica is more frequent in patients with dental abnormalities. However, Tassoker et al. [19] found no statistically significant correlation between sella bridging on panoramic radiographs and PDC, which is in accordance with the present study. Similar results were reported by Ortiz et al. [26], who analyzed 76 CBCT scans and found no significant correlation between maxillary palatal canine impaction and sella turcica bridging.

No studies have been found reporting on a possible correlation between sella turcica abnormalities and dental age. In the present study, sellar bridging was not related to chronological or dental age. However, sellar abnormalities were correlated with the difference between dental and chronological age. For the assessment of age development with Demirjian’s method, the patients included had to be within the developmental period of the dentition. Caderberg et al. [11] found a correlation between age and the degree of calcification of the ligaments sellar interclinoid and petroclinoid. Similarly, Arcos-Palomino and Ustrell-Torent [29] reported that sella turcica bridging is significantly age dependent, whereas in the present study, no significant correlation was found. The reason for diverse results is probably age range in the studies discussed, which was 6–15 years in the present study and 10–50 years in the study by Arcos-Palomino and Ustrell-Torent [29].

In the study by Różyło-Kalinowska et al. [33], dental age was significantly delayed both in cases of palatal and buccal impaction of upper canines. It must be emphasized that only impacted and not ectopically erupted teeth were taken into consideration in the cited study. A significant difference in dental development between patients with and without impacted maxillary canines has been reported by Lovgren et al. [41] as well. Becker and Chaushu [32] analyzed dental age in subjects with palatally displaced maxillary canines; 50% of subjects were characterized by a late-developing dentition, whereas subjects with normally positioned canines and those with buccally displaced canines had a normal timeline of dentition. This finding supports the hypothesis of a genetic etiology of palatal ectopia in maxillary canines [32].

The possible limitations of this study may be attributed to the number of radiographs examined; geographic limitations—all the radiographs came from the same research center; the inclusion of a limited number of cases due to the low percentage of dental abnormalities in the general population; and the accuracy of dental age assessment methods.

## 5. Conclusions

1. There is an association between sella turcica abnormalities and delayed dental age.
2. Dental abnormalities are more frequent in patients with sella turcica abnormalities.
3. Dental abnormalities are less frequent in subjects with sella turcica bridging in comparison with other sella turcica abnormalities.

## Figures and Tables

**Table 1:** Distribution of the study group according to chronologic and dental age.

Age (Months)				Dental Age (Months)			Difference between Chronologic and Dental Age		
	Males	Females	Total	Males	Females	Total	Males	Females	Total
mean	121.24	125.47	123.67	136.69	145.92	141.95	−15.45	−20.45	−18.28
median	117.93	120.43	120.40	134.40	140.40	137.40	−16.47	−19.97	−17.00
SD	24.24	23.03	23.53	24.31	24.11	24.51	−0.08	−1.08	−0.98
Q1	102.43	109.10	107.58	121.80	128.40	128.40	−19.37	−19.30	−20.83
Q3	136.97	141.27	140.50	147.60	162.00	155.40	−10.63	−20.73	−14.90
Min	80.10	87.10	80.10	92.40	98.40	92.40	−47.27	−53.33	−53.33
Max	179.10	183.20	179.10	192.00	192.00	192.00	8.77	0.00	8.77

**Table 2:** Sella turcica types in the present study (%, *n* = 206).

Sella Type	Males *n* = 95	Females *n* = 111	Total *n* = 206 (%)
SN	52.63% (50)	45.05% (50)	48.54% (100)
SA	0% (0)	2.70% (3)	1.46% (3)
SB	7.37% (7)	0.90% (1)	3.88% (8)
SC	10.53% (10)	11.71% (13)	11.17% (23)
SD	7.37% (7)	12.61% (14)	10.19% (21)
SE	4.21% (4)	1.80% (2)	2.91% (6)
SF	4.21% (4)	11.71% (13)	8.25% (17)
SG	7.37% (7)	3.60% (4)	5.34% (11)
SH	3.16% (3)	2.70% (3)	2.91% (6)
SI	2.11% (2)	2.70% (3)	2.43% (5)
SJ	1.05% (1)	4.50% (5)	2.91% (6)

**Table 3:** Dental abnormalities in the study and control groups.

Dental Abnormalities	Sella Turcica Type			
	Control Group: SN (*n* = 100)	Study Group with Sella Turcica Abnormalities (*n* = 106)		Total (*n* = 206)
		Bridge: SA. SB. SC (*n* = 34)	Other: SD–SJ (*n* = 72)	
PDC (palatally displaced canine)	1.00% (*n* = 1)	2.94% (*n* = 1)	1.39% (*n* = 1)	1.46% (*n* = 3)
Hypodontia (1 or more teeth)	1.00% (*n* = 1)	2.94% (*n* = 1)	12.50% (*n* = 9)	5.34% (*n* = 11)
Hyperdontia	0	2.94% (*n* = 1)	4.17% (*n* = 3)	1.94% (*n* = 4)
Transposition	0	0	1.39% (*n* = 1)	0.49% (*n* = 1)
Impacted tooth	1.00% (*n* = 1)	2.94% (*n* = 1)	2.78% (*n* = 2)	1.94% (*n* = 4)
Total	3.00% (*n* = 3)	11.76% (*n* = 4)	22.22% (*n* = 16)	11.17% (*n* = 23)

## Data Availability

All the raw data are available from corresponding authors upon reasonable request.
